# Supplementary material for: Species Dependent Metabolism of a Covalent nsP2 Protease Inhibitor with in Vivo Anti-alphaviral Activity
Source: bioRxiv. 2025 Jan 16:2025.01.13.632788. Preprint. [Version 1] doi: 10.1101/2025.01.13.632788 (PMC11761022; doi:10.1101/2025.01.13.632788)

Supporting Information

Species Dependent Metabolism of a Covalent nsP2 Protease Inhibitor with *in Vivo* Anti-alphaviral Activity.

Mohammad Anwar Hossain[†,#], Abigail K. Mayo[‡], Anirban Ghoshal[†,#], Sharon A. Taft-Benz[§,#], Elizabeth J. Anderson[§,#], Noah L. Morales[§,#], Katia D. Pressey[§,#], Ava M. Vargason[▽,#], Kim L. R. Brouwer[‡], Nathaniel J. Moorman[§,#], Mark T. Heise[⊥,§,#], and Timothy M. Willson[†,#,*]

[†]Structural Genomics Consortium, UNC Eshelman School of Pharmacy, University of North Carolina at Chapel Hill, Chapel Hill, NC 27599, USA.

[#]READDI AViDD Center, University of North Carolina at Chapel Hill, Chapel Hill, NC 27599, USA.

[‡]Division of Pharmacotherapy and Experimental Therapeutics, UNC Eshelman School of Pharmacy, University of North Carolina at Chapel Hill, Chapel Hill, NC 27599, USA

[§]Department of Microbiology and Immunology, University of North Carolina at Chapel Hill, Chapel Hill, NC, 27599, USA

[▽]Eshelman Innovation, UNC Eshelman School of Pharmacy, University of North Carolina at Chapel Hill, Chapel Hill, NC 27599, USA

[⊥]Department of Genetics, University of North Carolina at Chapel Hill, Chapel Hill, NC, 27599, USA

*Correspondence: tim.willson@unc.edu

Table of Contents

	Description	Page
Table S1	Plasma Protein Binding of **1**	S2
Table S2	Equations for Allometric Scaling	S3
Table S3	Blood to Plasma Ratios of **1**	S4
Figure S1	MS fragmentation of metabolites M1–18 from mouse plasma	S5–13
Figure S2	MS fragmentation of metabolites M1–22 from hepatocytes	S14–21
File S1	Pharmacokinetic data – mouse	
File S2	Pharmacokinetic data – rat, dog, monkey	

Table S1. Plasma Protein Binding of 1[a]

Species	Protein binding (%)	f_u
Mouse	82	0.18
Rat	89	0.11
Dog	80	0.20
Human	86	0.14

[a] Plasma protein binding was determined by equilibrium dialysis. For monkey, the human value used.

Table S2. Equations for Allometric Scaling

Calculated Value	Equation
Systemic Clearance	$CL_s = \frac{F \times X_0}{AUC_{PO}}, where\ F = \frac{AUC_{PO}}{AUC_{IV}} \times \frac{X_{0,IV}}{X_{0,PO}}$
Intrinsic Clearance[a]	$CL_{int,in\ vivo} = \frac{hepatocellularity\ (\frac{10^6 cells}{g\ liver}) \times \frac{liver\ weight\ (g)}{kg\ body\ weight\ (kg)} \times CL_{int,in\ vitro}(\mu L/min/10^6 cells)}{fu_{in\ vitro}}$
IVIVE Scaling Factor[b]	$Scaling\ factor = hepatocellularity\ (10^6 cells/g\ liver) \times liver\ weight\ (g/kg) \times \frac{1\ mL}{1000\ uL}$
Blood Clearance	$CL_{int,blood} = CL_{int,plasma} \times \frac{C_{plasma}}{C_{blood}}$
Hepatic Clearance	$CL_h = \frac{Q_{blood} \times fu \times CL_{int,in\ vivo}}{Q_{blood} + fu \times CL_{int,in\ vivo}}$
Hepatic Extraction	$E_{hepatic} = \frac{\frac{Q_{blood} \times fu \times CL_{int,in\ vivo}}{Q_{blood} + fu \times CL_{int,in\ vivo}}}{Q_{blood}} = \frac{CL_h}{Q_{blood}}$
i.v. Infusion Rate	$k_0 = infusion\ rate = C_{ss} \times CL$

[a]Calculations assumed $fu_{in\ vitro} = 1$

[b]Values from Petersson, C., Zhou, X., Berghausen, J., Cebrian, D., Davies, M., DeMent, K., & Baker, J. (2022). Current approaches for predicting human PK for small molecule development candidates: findings from the IQ human PK prediction working group survey. The AAPS journal, 24(5), 85. https://doi.org/10.1208/s12248-022-00735-9

Table S3. Blood to Plasma Ratios of 1

	Blood:plasma ratio ($K_{b/p}$)[a]
Rat	1.37
Monkey	1.41
Human	1.12

[a]For Mouse and Dog a nominal value of 1.0 was used in IVIVE calculations based on measured values in the other species.

Figure S1. MS fragmentation of metabolites M1–18 from mouse plasma

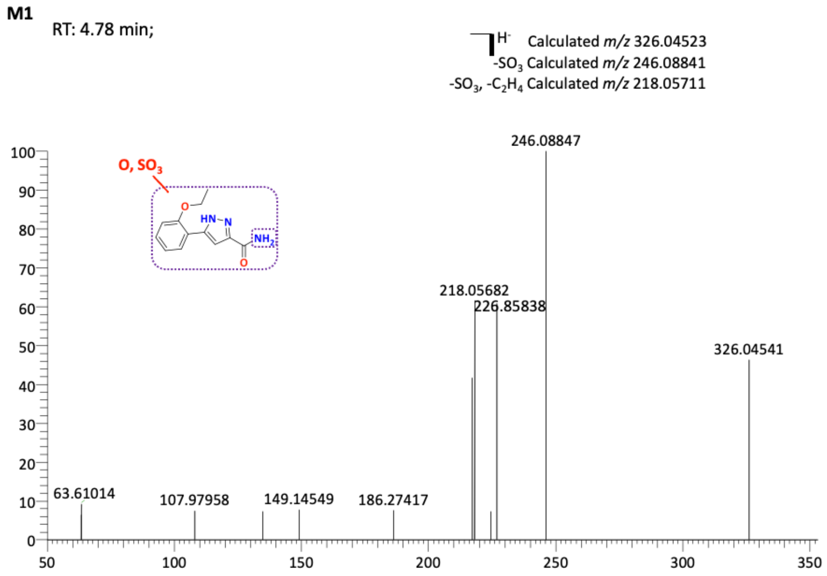

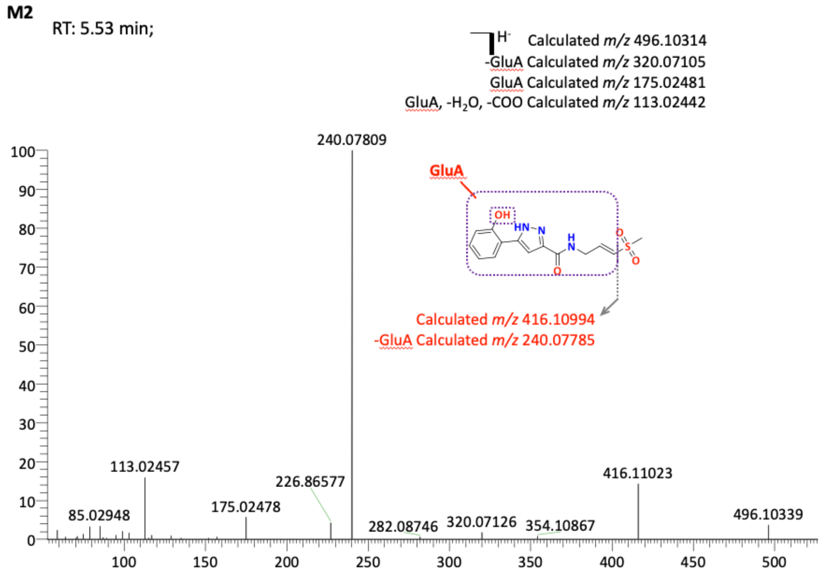

M3

RT: 5.60 min;

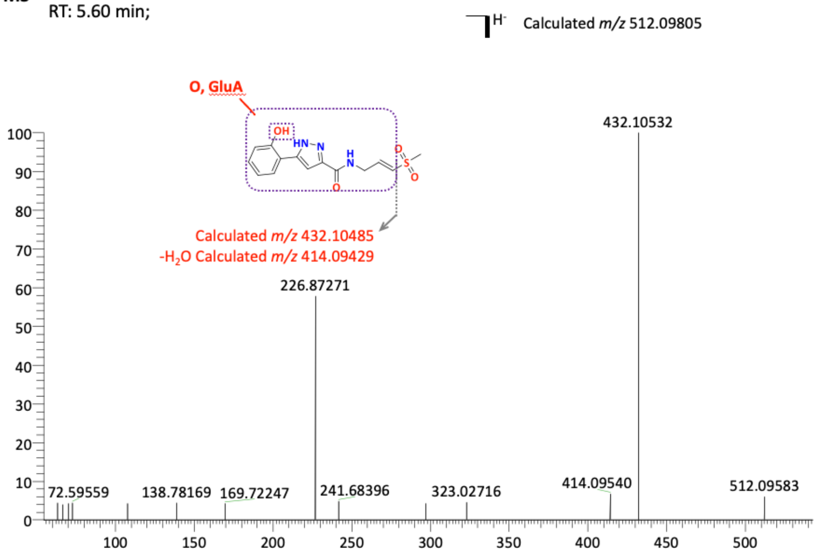

M4

RT: 5.62 min;

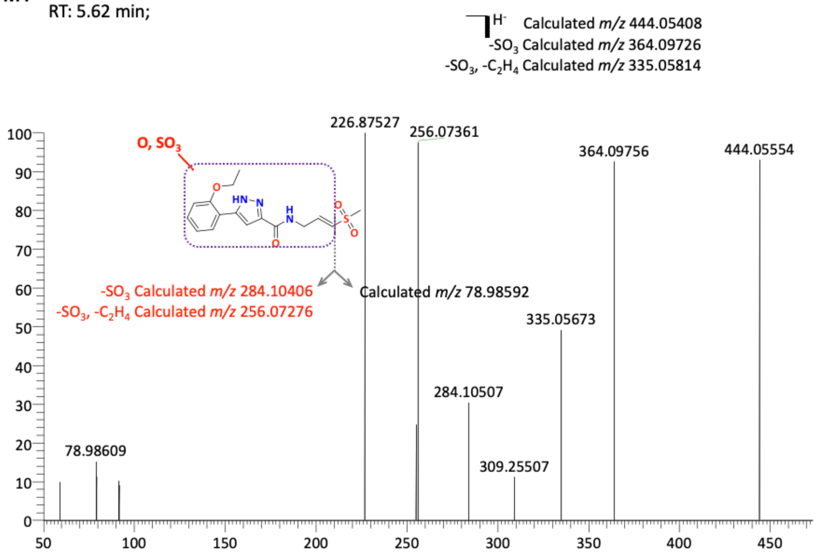

M5

RT: 5.72 min;

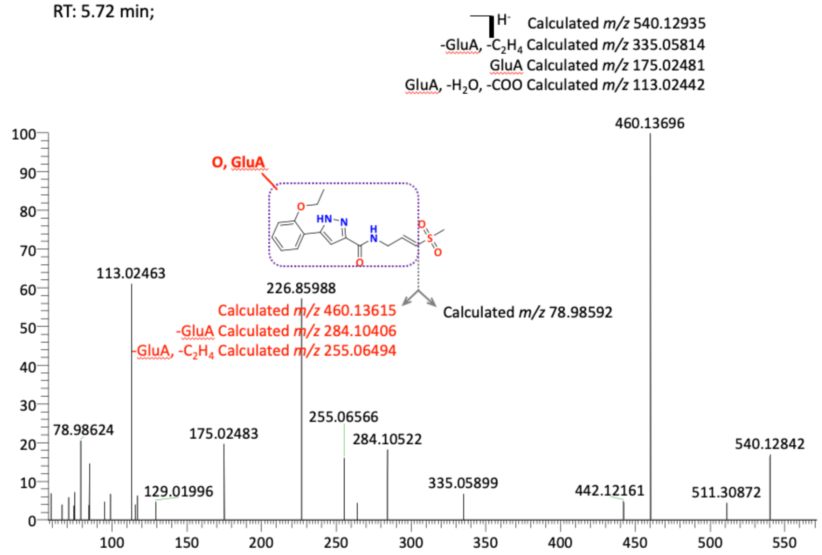

M6

RT: 6.06 min;

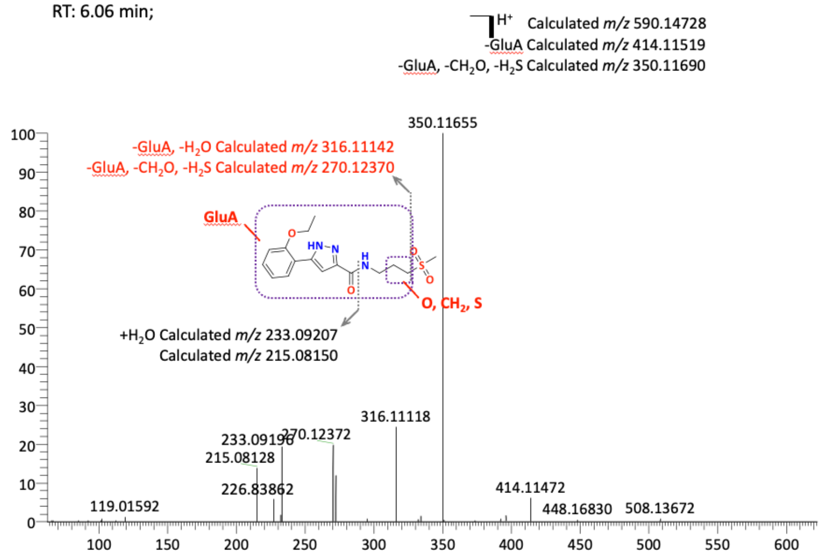

M7

RT: 6.16 min;

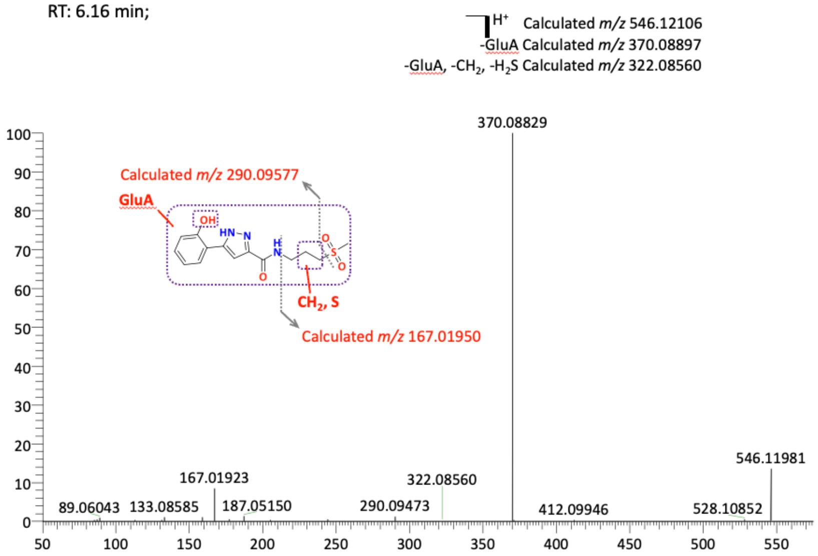

M8

RT: 6.29 min;

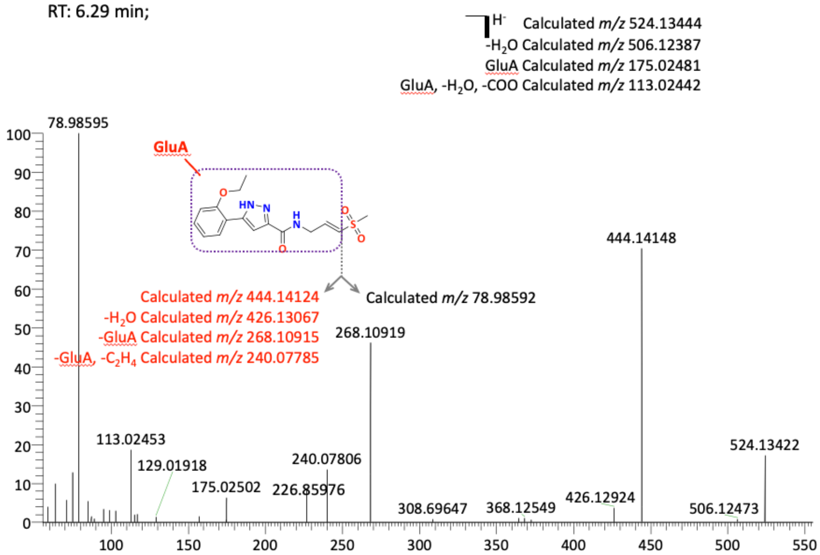

M9

RT: 6.50 min;

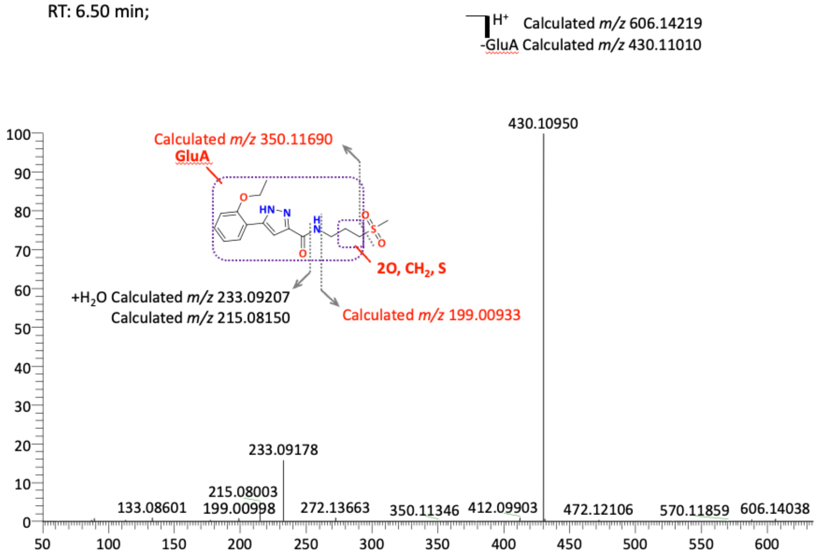

M10

RT: 6.70 min;

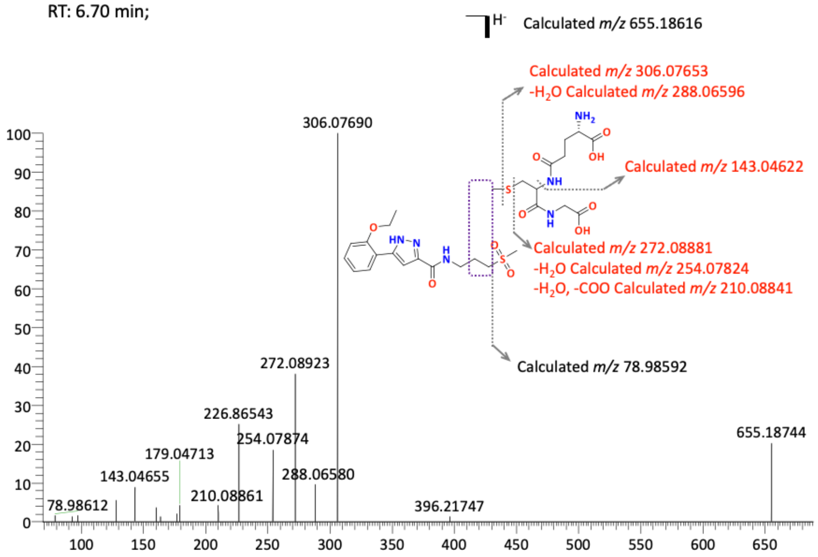

M11

RT: 6.74 min;

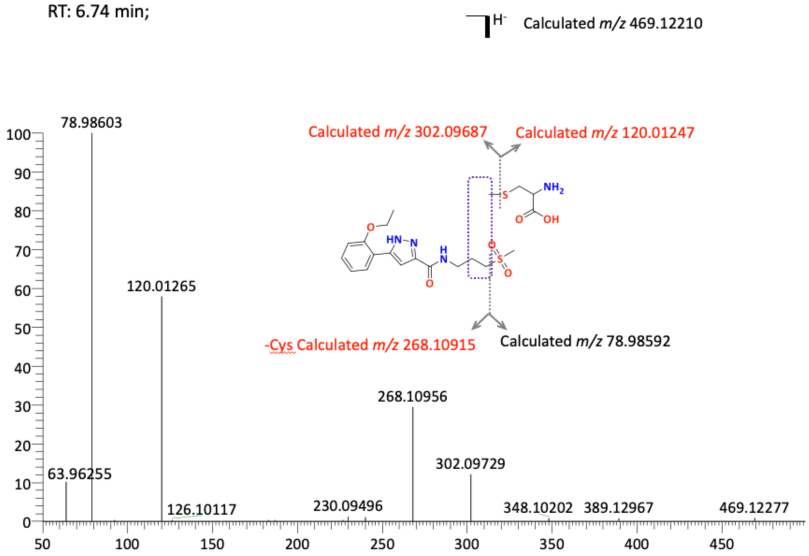

M12

RT: 6.81 min;

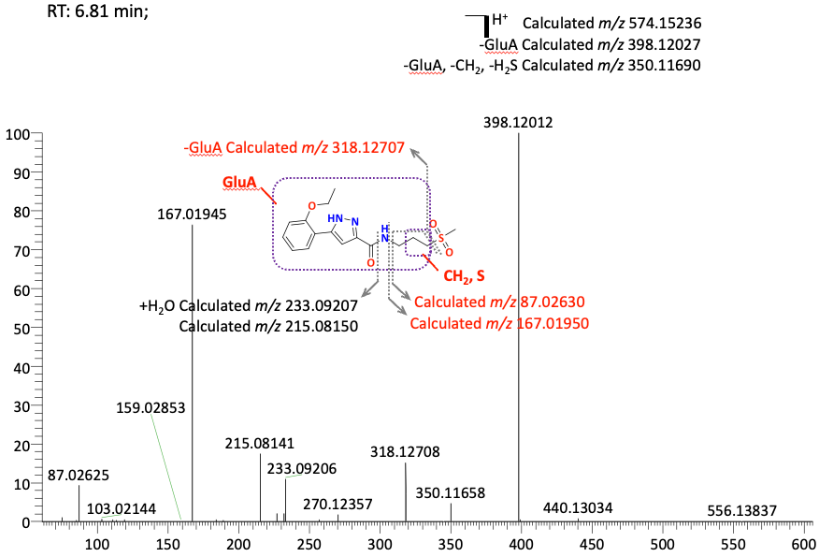

M13

RT: 7.02 min;

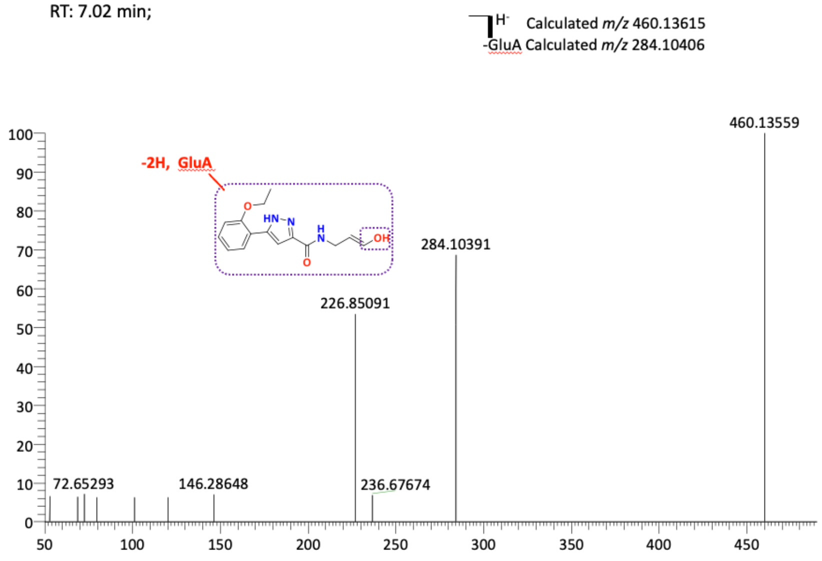

M14

RT: 7.49 min;

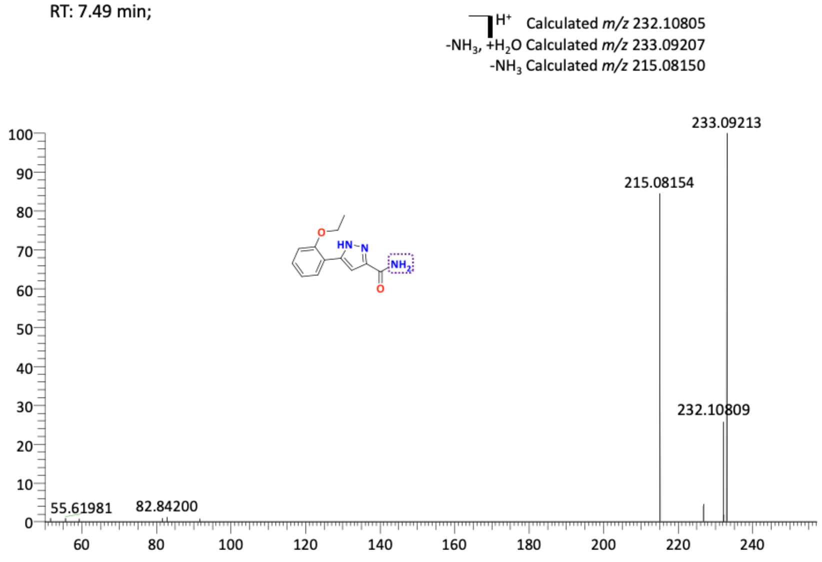

M15

RT: 7.75 min;

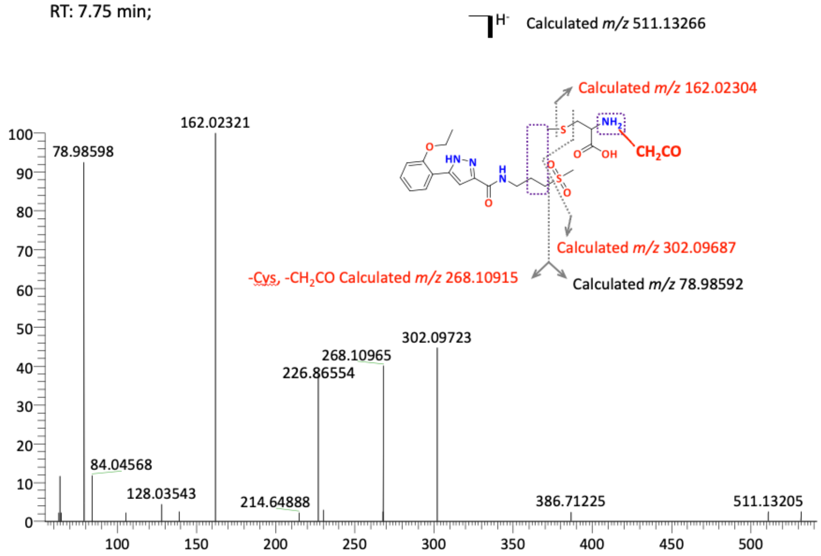

M16

RT: 8.06 min;

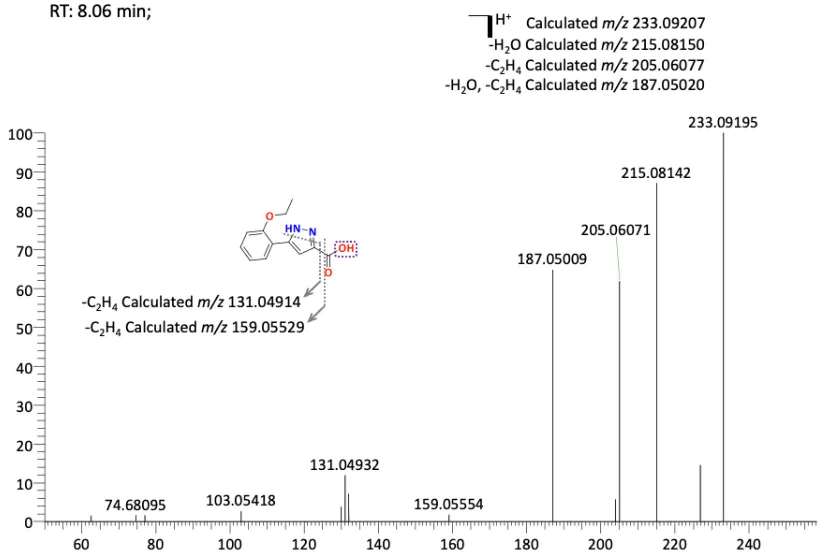

M17

RT: 8.34 min;

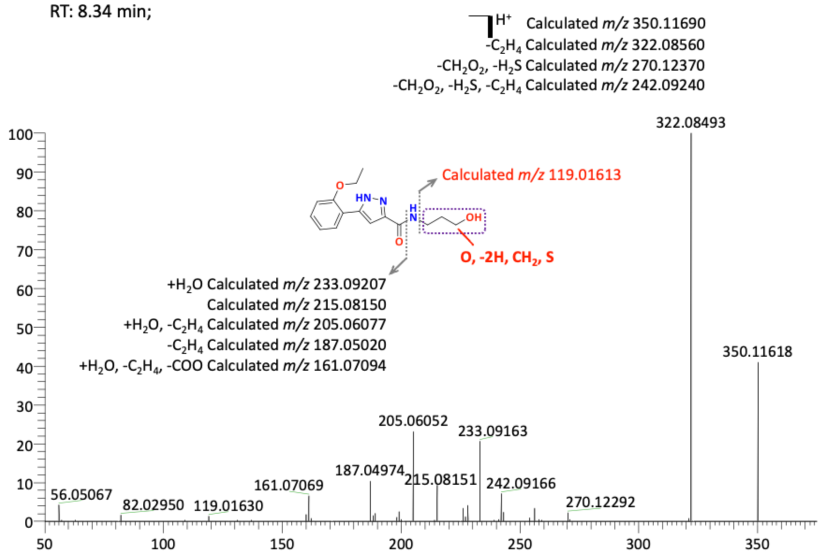

M18

RT: 8.97 min;

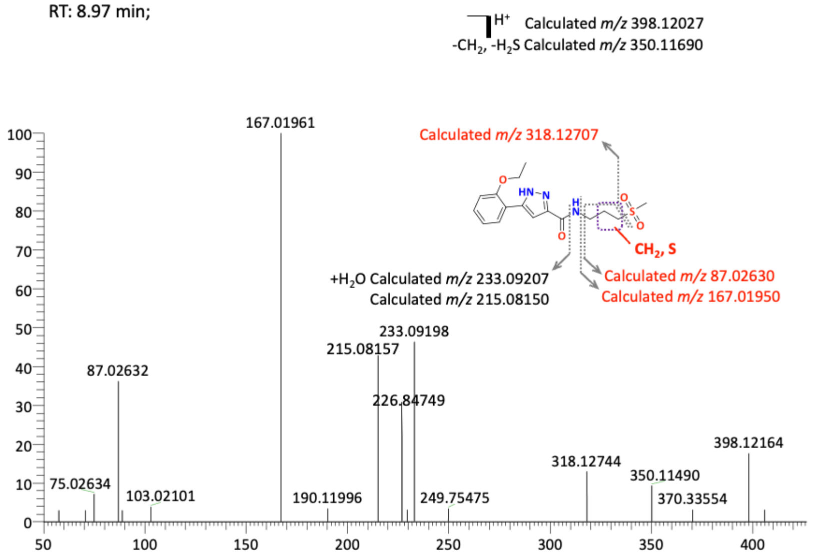

Figure S2. MS fragmentation of metabolites M1–22 from hepatocytes

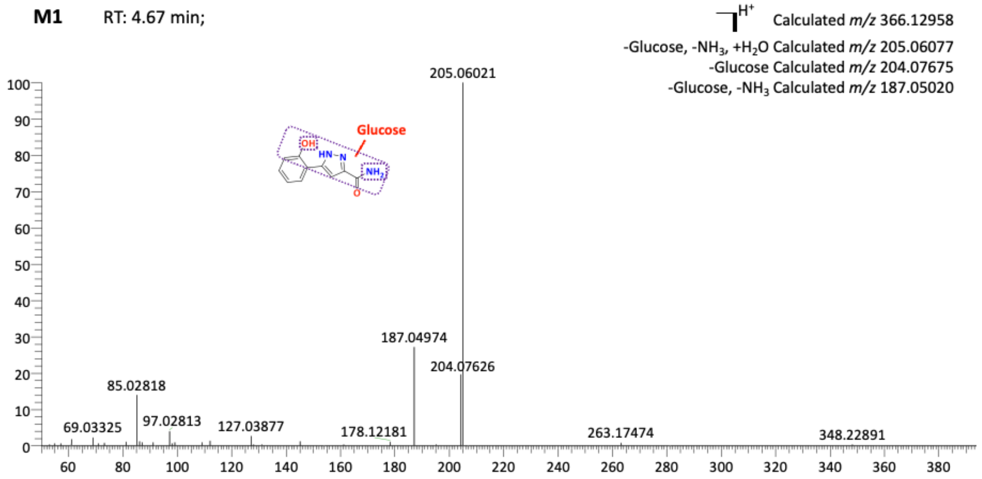

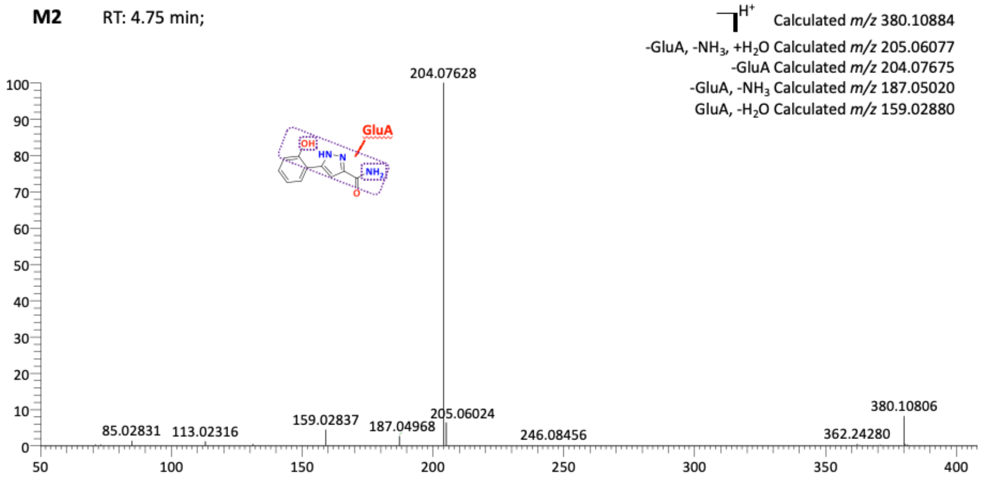

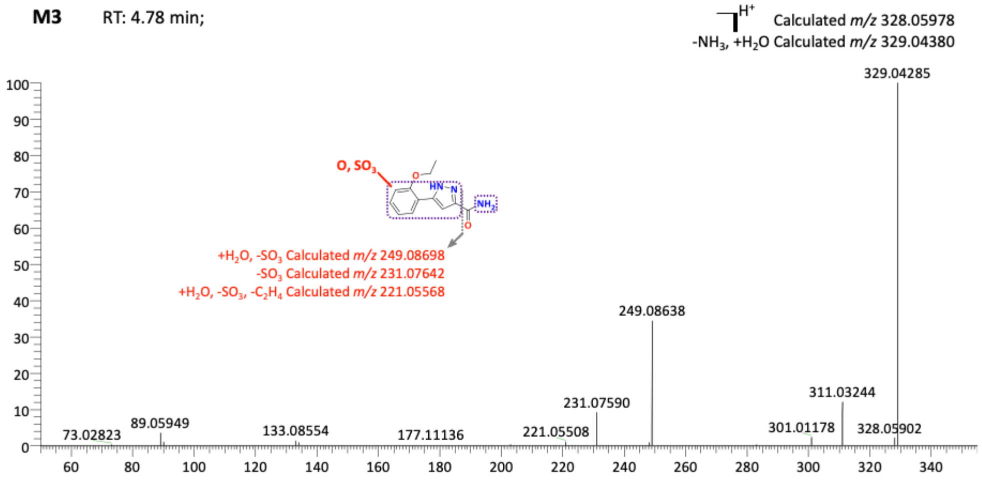

M4 RT: 5.54 min;

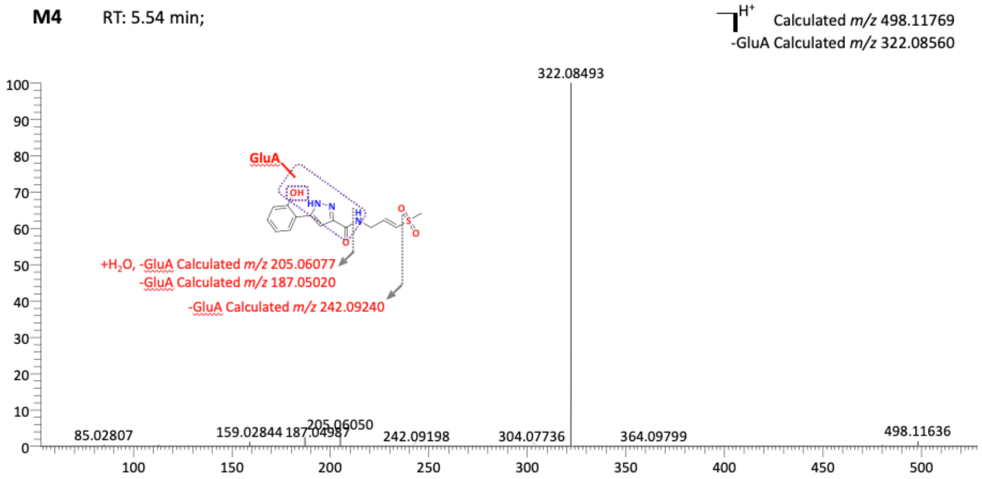

M5 RT: 5.60 min;

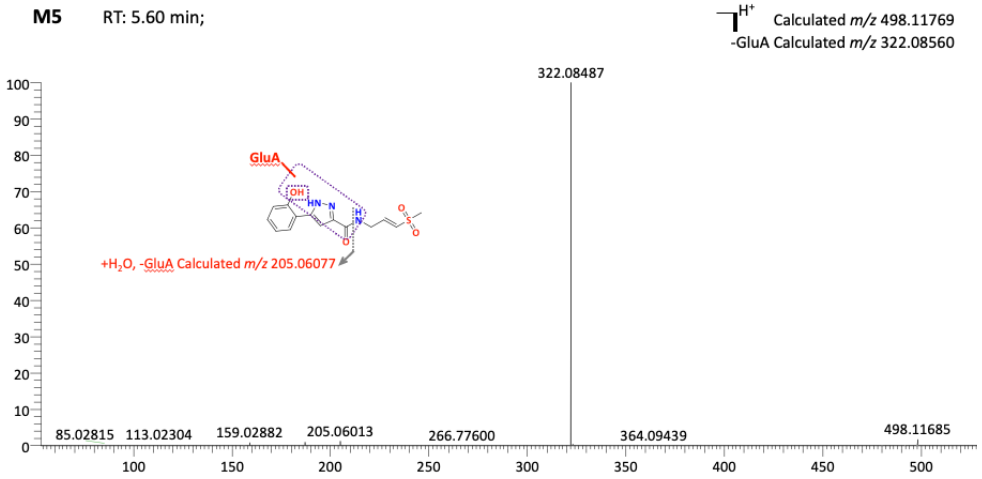

M6 RT: 5.61 min;

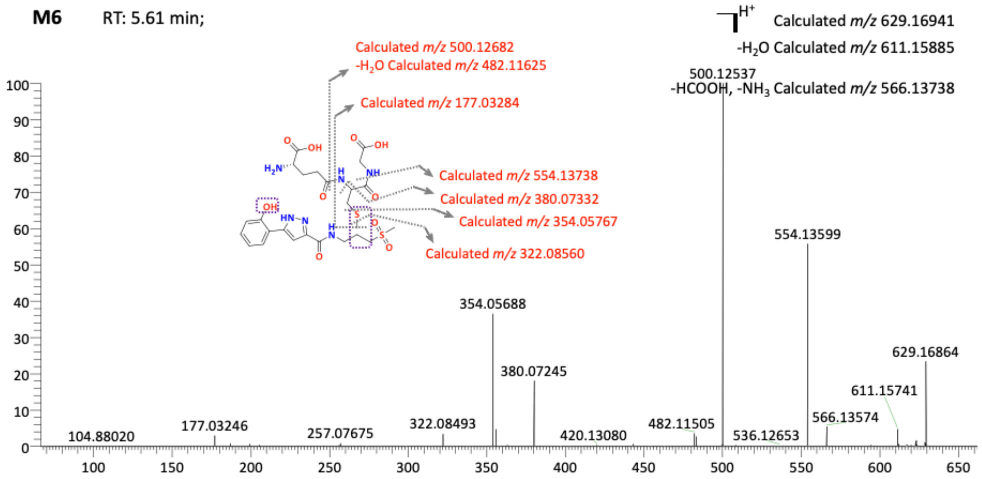

M7 RT: 5.76 min;

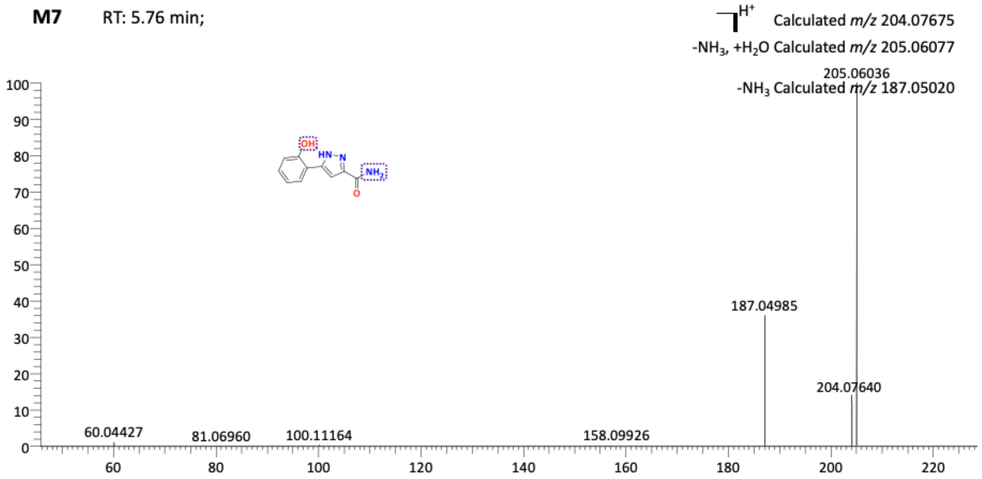

M8 RT: 6.73 min;

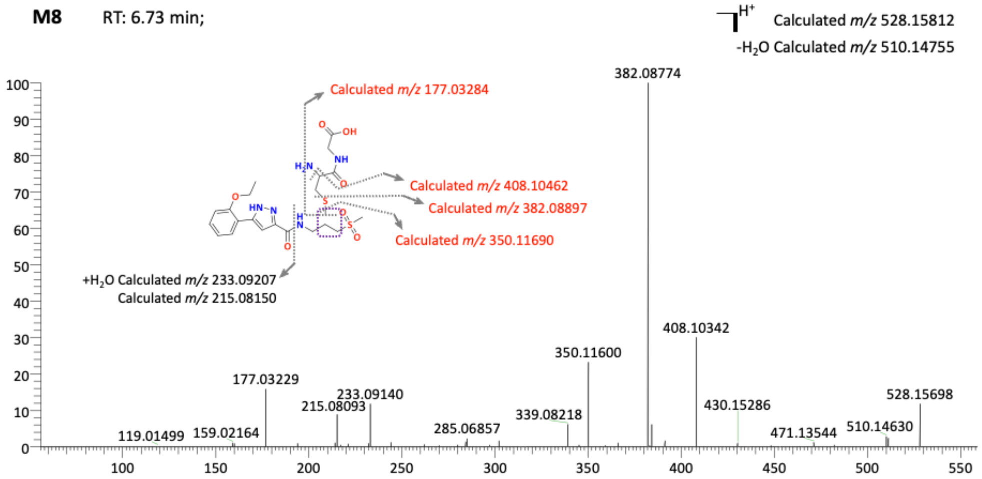

M9 RT: 6.74 min;

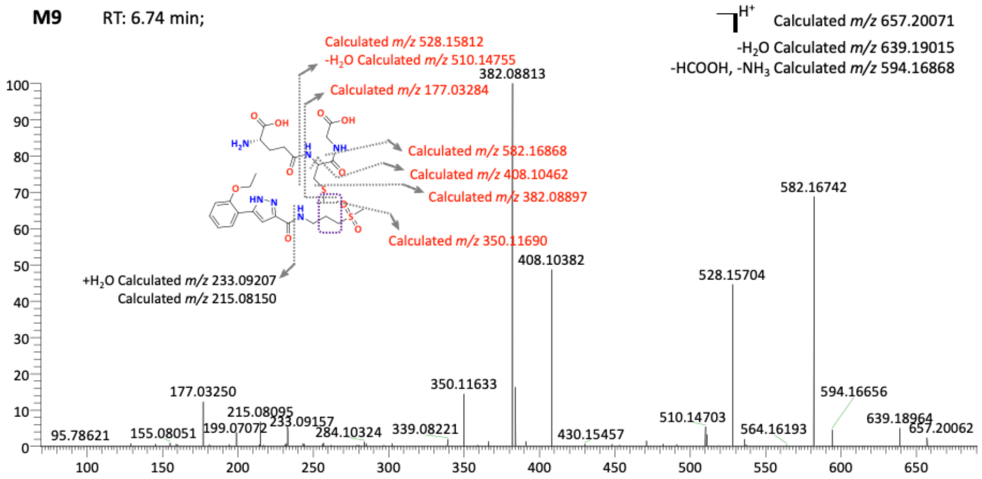

M10 RT: 6.76 min;

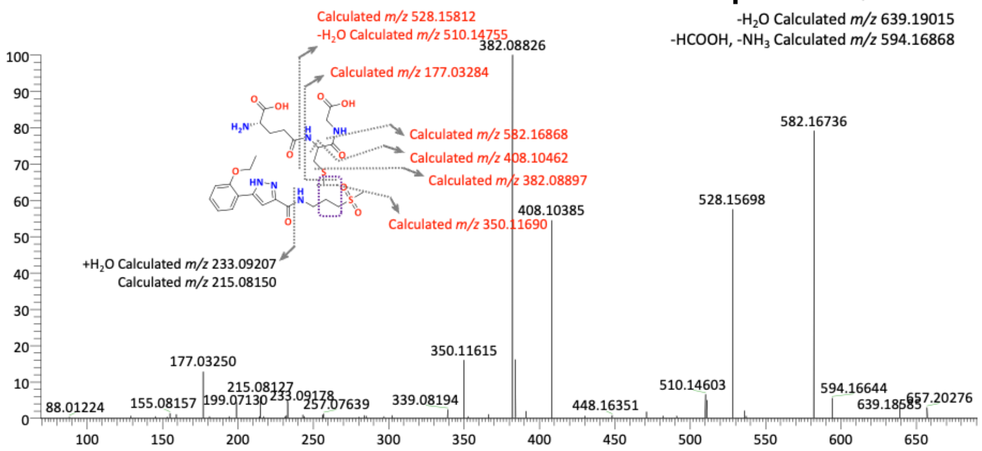

M11 RT: 6.79 min;

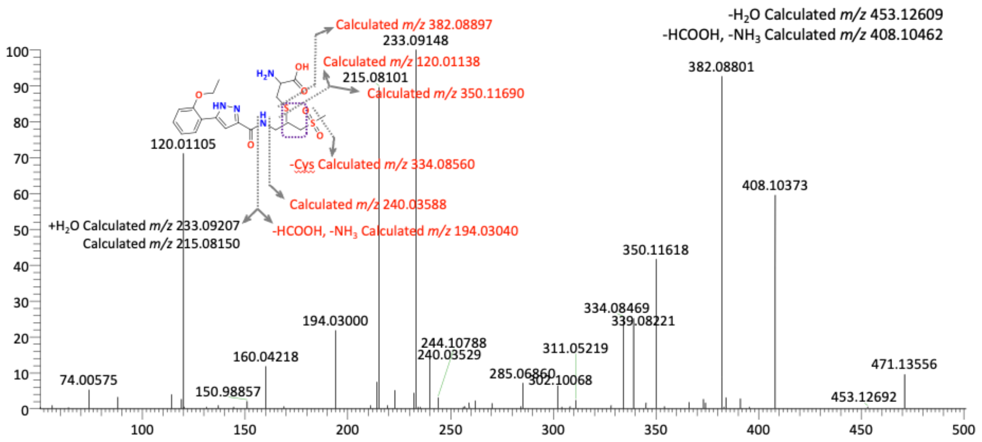

M12 RT: 6.97 min;

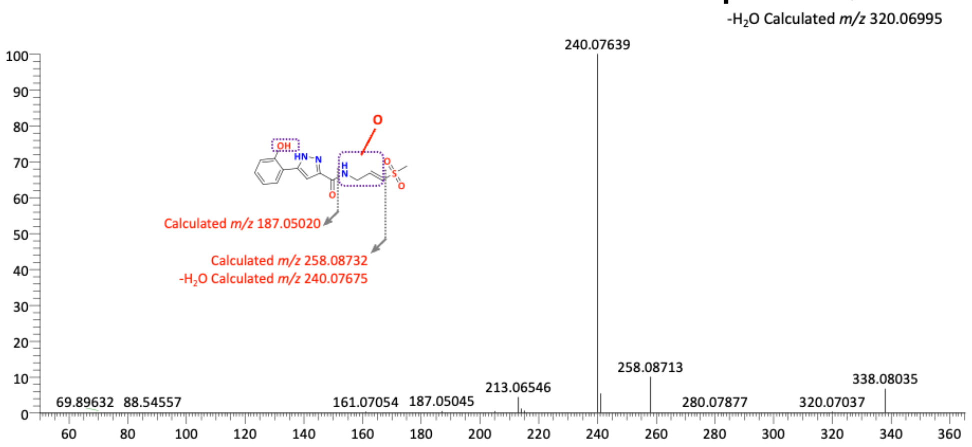

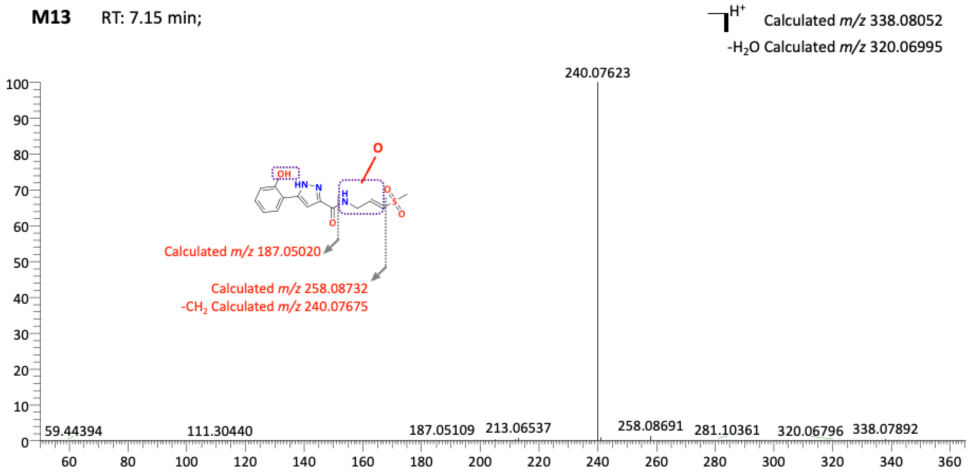
M13 RT: 7.15 min;
Calculated m/z 338.08052
-H2O Calculated m/z 320.06995
Calculated m/z 187.05020
Calculated m/z 258.08732
-CH2 Calculated m/z 240.07675
240.07623
59.44394
111.30440
187.05109
213.06537
258.08691
281.10361
320.06796
338.07892

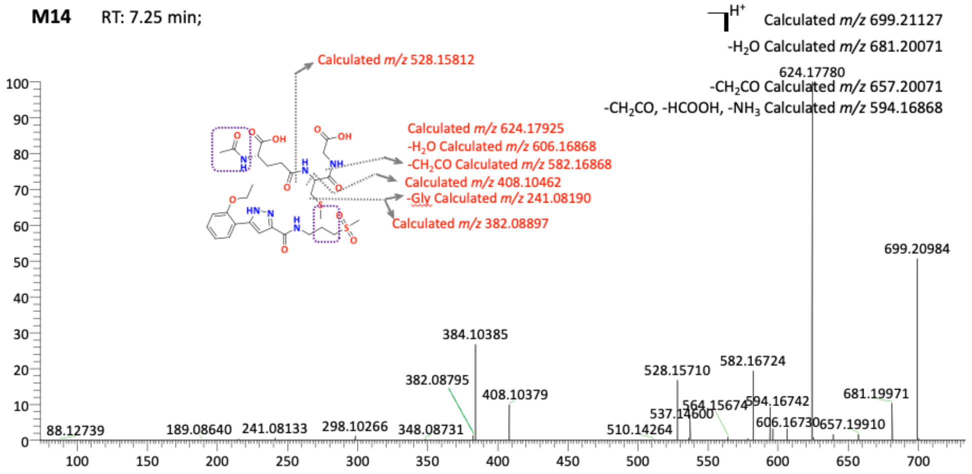
M14 RT: 7.25 min;
Calculated m/z 699.21127
-H2O Calculated m/z 681.20071
-CH2CO Calculated m/z 657.20071
-CH2CO, -HCOOH, -NH3 Calculated m/z 594.16868
Calculated m/z 528.15812
Calculated m/z 624.17925
-H2O Calculated m/z 606.16868
-CH2CO Calculated m/z 582.16868
Calculated m/z 408.10462
-Gly Calculated m/z 241.08190
Calculated m/z 382.08897
624.17780
699.20984
384.10385
382.08795
408.10379
528.15710
582.16724
537.14600
564.15674
594.16742
606.16730
657.19910
681.19971
510.14264
88.12739
189.08640
241.08133
298.10266
348.08731

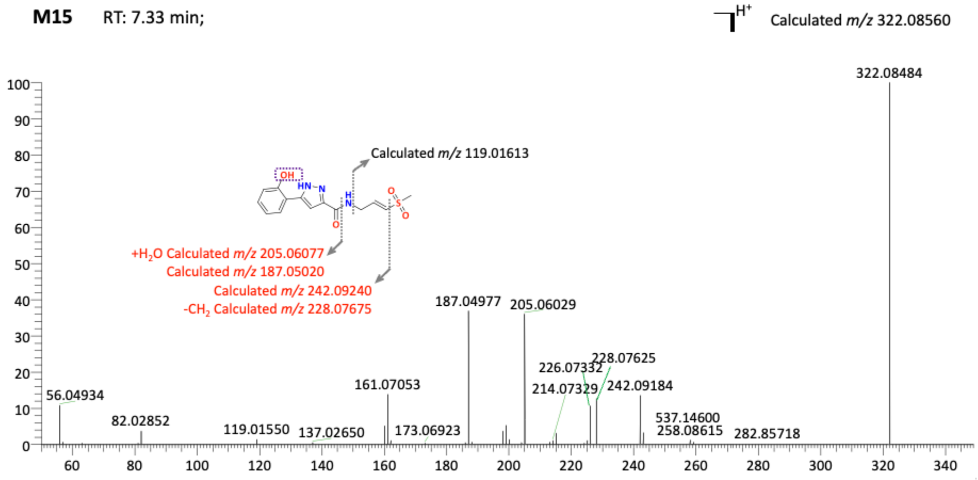
M15 RT: 7.33 min;
Calculated m/z 322.08560
Calculated m/z 119.01613
+H2O Calculated m/z 205.06077
Calculated m/z 187.05020
Calculated m/z 242.09240
-CH2 Calculated m/z 228.07675
322.08484
187.04977
205.06029
226.07332
228.07625
214.07329
242.09184
161.07053
56.04934
82.02852
119.01550
137.02650
173.06923
537.14600
258.08615
282.85718

M16 RT: 7.52 min;

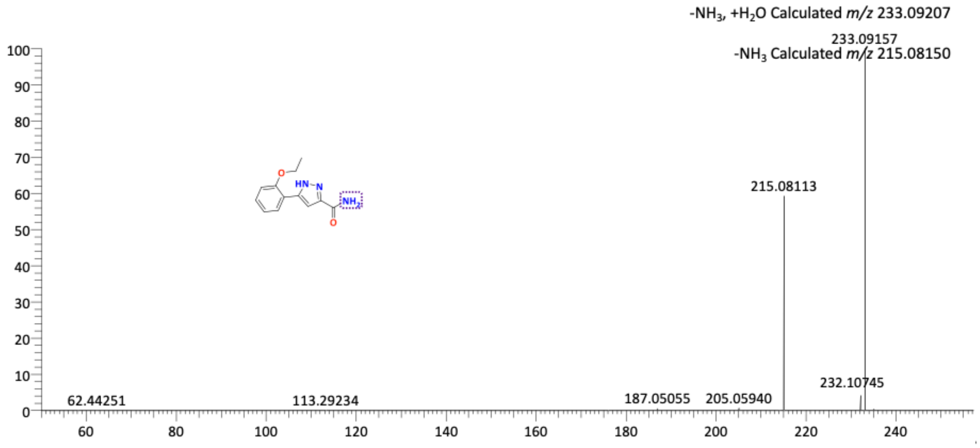

M17 RT: 7.79 min;

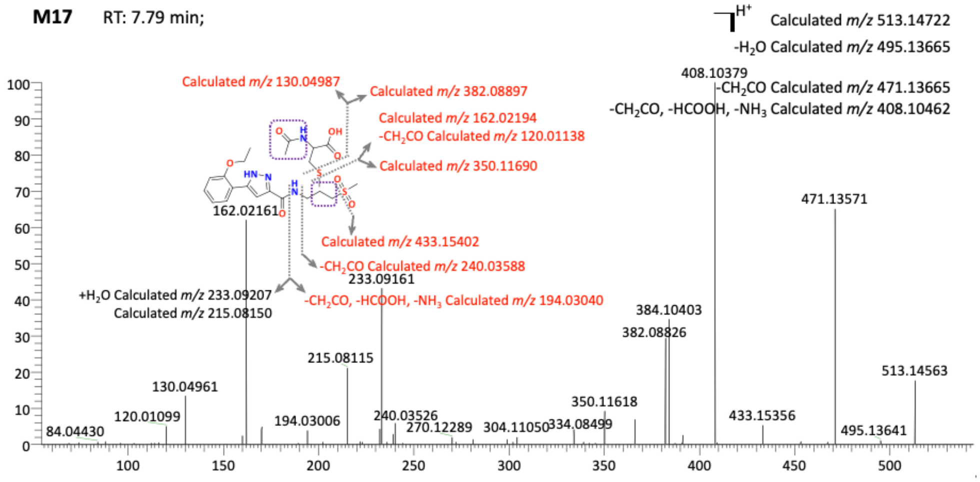

M18 RT: 7.98 min;

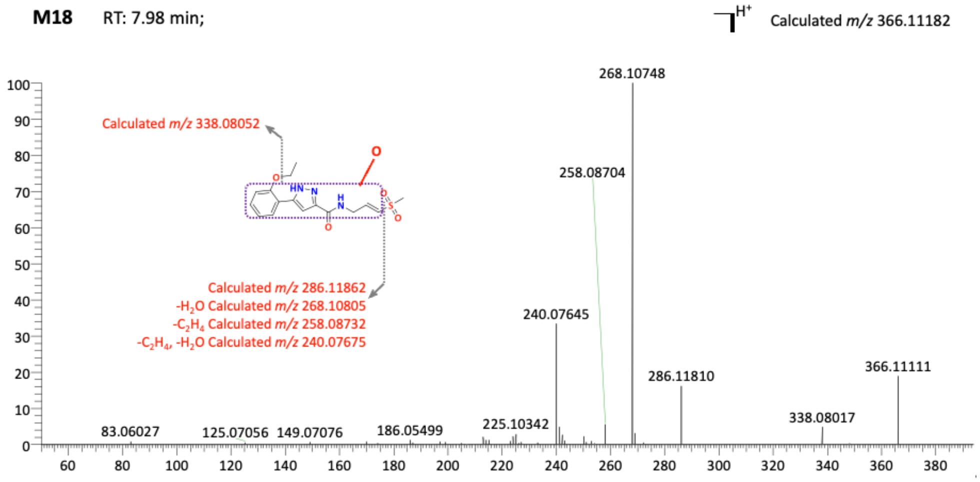

M19 RT: 8.03 min;

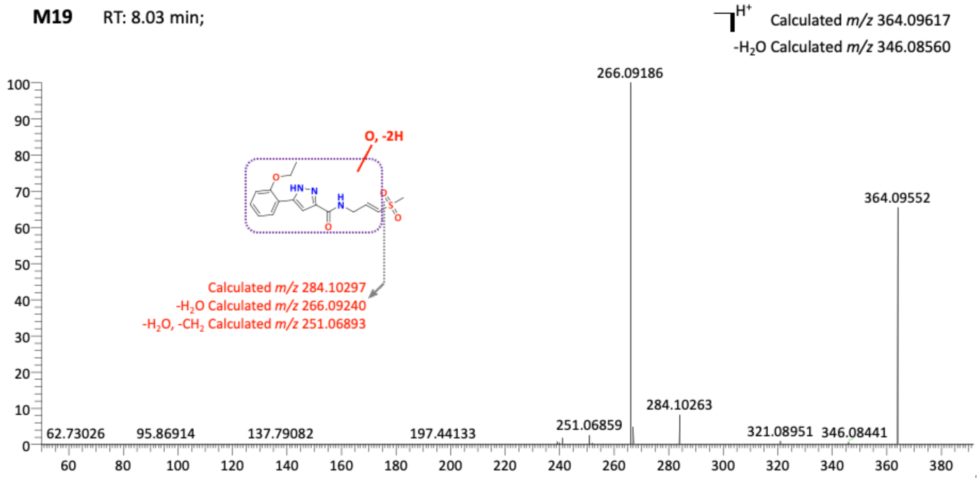

M20 RT: 8.08 min;

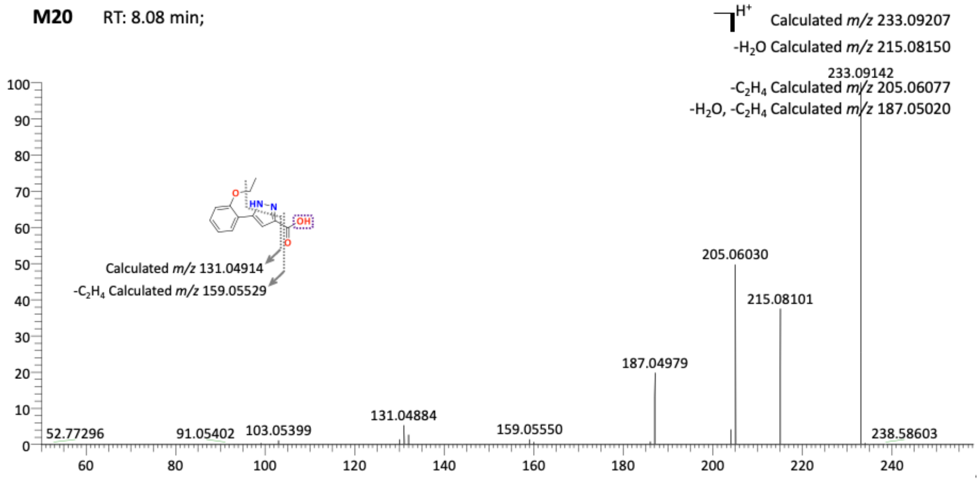

M21 RT: 8.39 min;

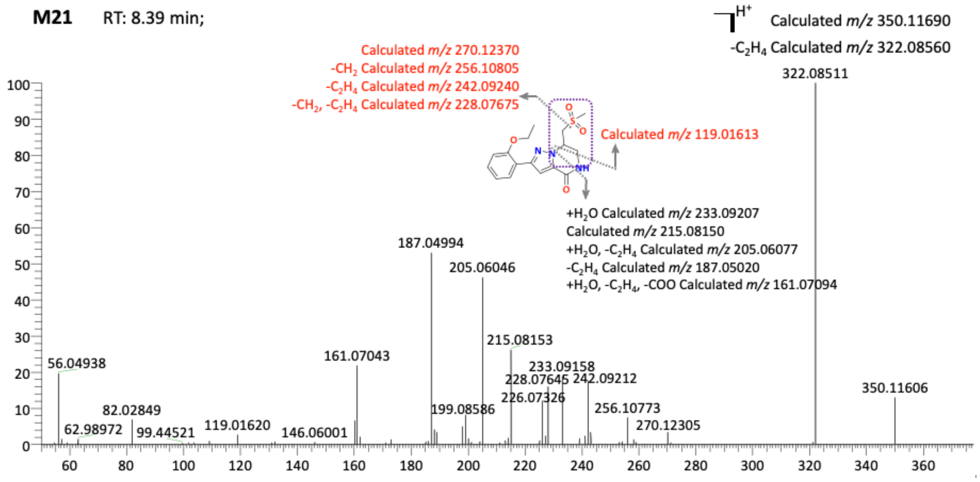

M22 RT: 8.50 min;

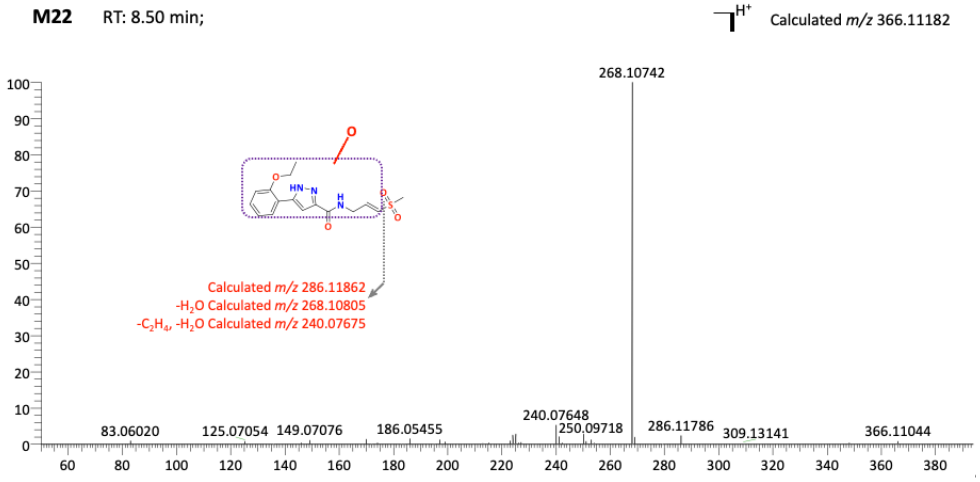

Supplement: Supplement 1 [file media-1.pdf]
